# TetraSOD^®^, a Unique Marine Microalgae Ingredient, Promotes an Antioxidant and Anti-Inflammatory Status in a Metabolic Syndrome-Induced Model in Rats

**DOI:** 10.3390/nu14194028

**Published:** 2022-09-28

**Authors:** Katherine Gil-Cardoso, Josep M. Del Bas, Antoni Caimari, Carmen Lama, Sonia Torres, Lalia Mantecón, Carlos Infante

**Affiliations:** 1 Eurecat, Centre Tecnològic de Catalunya, Technological Unit of Nutrition and Health, 43204 Reus, Spain; 2 Eurecat, Centre Tecnològic de Catalunya, Biotechnology Area, 43204 Reus, Spain; 3 Fitoplancton Marino S.L., Dársena Comercial, S/N (Muelle Pesquero), 11500 Cádiz, Spain

**Keywords:** TetraSOD^®^, *Tetraselmis chuii*, microalgae, metabolic syndrome, oxidative stress, inflammation

## Abstract

Increased oxidative stress has been linked to the pathogenic process of obesity and can trigger inflammation, which is often linked with the risk factors that make up metabolic syndrome (MetS), including obesity, insulin resistance, dyslipidaemia and hypertension. TetraSOD^®^, a natural marine vegan ingredient derived from the microalgae *Tetraselmis chuii* that is high in the antioxidant enzymes superoxide dismutase (SOD), catalase (CAT), and glutathione peroxidase (GPx) has recently demonstrated in vitro increased activity of these key antioxidant enzymes. In the present study, the potential bioactive effects of three dietary dosages of TetraSOD^®^ in enhancing antioxidant and anti-inflammatory mechanisms to combat the metabolic disturbances that compose MetS were assessed in rats given a cafeteria (CAF) diet. Chronic supplementation with 0.17, 1.7, and 17 mg kg^−1^ day^−1^ of TetraSOD^®^ for 8 weeks ameliorated the abnormalities associated with MetS, including oxidative stress and inflammation, promoting endogenous antioxidant defence mechanisms in the liver (GPx and GSH), modulating oxidative stress and inflammatory markers in plasma (NOx, oxLDL and IL-10), and regulating genes involved in antioxidant, anti-inflammatory and immunomodulatory pathways in the liver, mesenteric white adipose tissue (MWAT), thymus, and spleen. Overall, TetraSOD^®^ appears to be a potential therapeutic option for the management of MetS.

## 1. Introduction

Metabolic syndrome (MetS) is defined as a cluster of interconnected risk factors that include insulin resistance, obesity, dyslipidaemia and hypertension [1]. MetS is associated with an increased risk of cardiovascular disease (CVD) and type-2 diabetes (T2D), the two primary causes of mortality worldwide [2]. The World Health Organization estimated that CVDs represent the leading cause of death globally, accounting for an estimated 17.9 million lives each year, while diabetes causes another 1.6 million deaths [3]. Different studies carried out in both humans [4,5] and animals [6,7,8,9,10] have shown that obesity and its associated metabolic disturbances, such as hyperglycaemia, hyperlipidaemia and insulin resistance, are linked to a drop in the antioxidant capacity in blood and/or several tissues. In turn, this oxidative stress can lead to various health complications often linked to obesity, such as endothelial dysfunction, nonalcoholic fatty liver disease, microvascular complications and nephropathy [11]. The main contributors to these aforementioned deleterious effects on antioxidant defence are an increased intake of fat and sugars at the expense of fruits and vegetables, which are rich sources of vitamins, minerals and polyphenols, and the elevated high reactive oxygen species (ROS) production that characterize the obese state [11]. Therefore, all of these concerns highlight the need to identify innovative strategies to prevent or ameliorate this multifactorial disorder.

Among these approaches, the use of antioxidants has gained increasing interest. A growing body of scientific evidence has suggested a potential therapeutic role of supplementation with dietary antioxidants, such as selenium or vitamins, in body weight loss and in several obesity-related disorders [5,12,13,14,15]. Recently, other attractive strategies have emerged to combat the oxidative stress and the chronic inflammatory status associated with MetS, such as the administration of polyphenol extracts [6,8,16] and food bioactive compounds rich in antioxidant enzymes [17,18,19,20], which have longer lasting effects compared to mere metabolites [7]. Supplementation with *Syzygium cumini* seeds enriched in gallic acid, ellagic acid and epicatechin presented several beneficial effects in the liver of rats fed a cafeteria (CAF) diet, as a model of high-fat (HF) and high-carbohydrate (HC) diet [6]. The observed effects include the prevention of inflammatory cell infiltration and fat deposition and fibrosis, decreased hepatic thiobarbituric acid reactive substance (TBARS) levels, and increased expression of some antioxidant enzymes, such as superoxide dismutase (SOD) and catalase (CAT), and nonenzymatic antioxidant agents, such as glutathione (GSH) [6]. Moreover, hamsters fed a semipurified HF–high-cholesterol diet for 12 weeks and supplemented with a melon extract, characterized by high SOD levels, presented an improved metabolic profile in several aspects including: insulin resistance, dyslipidaemia, fat mass, atherosclerosis, steatosis, liver superoxide anion, cytochrome C activity, and lipid and protein oxidation products [17,19]. In addition, hamsters fed a CAF diet for 19 weeks that received a powdered melon SOD extract during the last month displayed reduced body weight, insulin resistance and hepatic oxidative stress and increased expression of the liver antioxidant defence proteins SOD, CAT, and GSH peroxidase (GPx) [7].

TetraSOD^®^ is a unique marine healthy and functional ingredient comprising 100% lyophilized powder of the microalgal species *Tetraselmis chuii* (*T. chuii*) strain CCFM03, a marine microalga with a history of use for food and nutraceutical applications around the world [21]. Fitoplancton Marino, S.L. (El Puerto de Santa María, Cádiz, Spain) has patented a process to produce high SOD *T. chuii* and *T. chuii* biomass containing increased SOD activity (the SOD activity is higher than 180 U/g) [22]. In addition, TetraSOD^®^ shows a balanced nutritional composition, containing vitamins, minerals, amino acids, essential fatty acids, polyphenols and pigments [22]. Some of these compounds are known to exhibit antioxidant and anti-inflammatory bioactivities, such as polyunsaturated fatty acids (PUFAs) or polyphenols, even acting with some kind of synergistic effect [23]. In this sense, PUFAs have been demonstrated to have powerful anti-inflammatory effects in in vitro and in vivo systems, reducing proinflammatory and/or increasing anti-inflammatory cytokine production [24,25,26], whereas polyphenols significantly contribute to the total antioxidant capacity of microalgal species and are recognized as important natural dietary antioxidants [27,28,29]. Recently, TetraSOD^®^ demonstrated a statistically significant induction of the primary antioxidant enzyme activities, SOD, GPx, and CAT, in muscle cells in vitro [22] and in vivo [30].

Diet-induced obesity (DIO) animal models are well-established methods to study obesity and MetS [31,32]. Among them, CAF diets more accurately reflect the variety of palatable energy-rich foods present in the Western diet connected to obesity and other metabolic alterations [33]. As in humans, the CAF diet also causes inflammation and oxidative stress in adipose tissue [9], liver [7,33] and intestine [34]. CAF diets induce obesity and other related metabolic disorders in a way that is more human-like than other HF diets [33,35].

In the present study, we hypothesized that supplementation of CAF-fed rats with TetraSOD^®^ ameliorates the oxidative stress and inflammation associated with MetS. To verify this hypothesis, we investigated the potential bioactive and corrective effects of three dietary doses of TetraSOD^®^ in improving the antioxidant and anti-inflammatory mechanisms to tackle the metabolic-associated disorders that comprise MetS in rats fed a CAF diet. As it is well established that obesity negatively affects immunity [36], a potential immunomodulatory effect of TetraSOD^®^ was also evaluated in the thymus and spleen as key tissues of the immune system.

## 2. Materials and Methods

### 2.1. Treatment

The product of interest is TetraSOD^®^, a green lyophilized powder comprising 100% of the marine microalga *Tetraselmis chuii* strain CCFM03. TetraSOD^®^ was provided by Fitoplancton Marino, S.L. (El Puerto de Santa María, Cádiz, Spain). This microalga is grown in controlled outdoor closed photobioreactors under photoautotrophic conditions that yield a product with SOD activity higher than 30,000 U/g dry weight.

### 2.2. Animals, Diets and Experimental Design

The Animal Ethics Committee of the Technological Unit of Nutrition and Health of EURECAT (Reus, Spain) and the Generalitat of Catalunya approved all procedures (9823). The experimental protocol complied with the ARRIVE guidelines, followed the ‘Principles of Laboratory Animal Care’ and was carried out in accordance with the EU Directive 2010/63/EU for animal experiments. Fifty 7-week-old male Sprague–Dawley rats weighing 200–230 g were used for this study. Rats were individually caged in the animal facility at 22 °C with a 12 h light/12 h dark cycle and were fed *ad libitum* a standard (STD) chow diet. After 7 days of acclimation, 40 rats were fed the CAF diet for 8 weeks to induce MetS, and 10 rats were maintained under an STD diet (Teklad Global 14% Protein Rodent Diet 2014, Envigo, Barcelona, Spain) over the same period. The caloric distribution of the STD (2.9 Kcal/g) and CAF (4.8 Kcal/g) diets [37] is specified in Table 1. Rats fed the CAF diet were allowed free access to milk with sugar (220 g/L; 100 mL), biscuit with pâté (13–14 g), biscuit with cheese (14–15 g), bacon (5–7 g) and muffins (7–8 g), as a variety of highly palatable, energy-dense, unhealthy human foods *ad libitum* plus carrots (6–8 g), an STD chow diet and water. At the end of the 8th week, to determine whether the CAF-fed animals had developed MetS features, blood samples were obtained under fasting conditions (8 h of diurnal fasting) by saphenous vein puncture to detect alterations in their biochemical parameters. In addition, body composition analyses were carried out in the 8th week. Afterwards, the CAF-fed rats were randomly divided into 4 groups depending on the treatment received over the remaining 8 weeks (*n* = 10 per group). This design resulted in a total of 5 experimental groups as follows: STD-C group, rats fed STD diet the first 8 weeks and STD diet plus vehicle the last 8 weeks of the study; CAF-C group, rats fed CAF diet the first 8 weeks and CAF diet plus vehicle the remaining 8 weeks; CAF + 0.17 group, rats fed CAF diet the first 8 weeks and CAF diet supplemented with 0.17 mg kg^−1^ body weight (bw) per day of TetraSOD^®^ the last 8 weeks; CAF + 1.7 group, rats fed CAF diet the first 8 weeks and CAF diet supplemented with 1.7 mg kg^−1^ bw per day of TetraSOD^®^ the last 8 weeks, and CAF + 17 group, rats fed a CAF diet the first 8 weeks and CAF diet supplemented with 17 mg kg^−1^ bw per day of TetraSOD^®^ the last 8 weeks. Water-diluted (1:1) skimmed condensed milk was used as a vehicle for the administration of TetraSOD^®^. Both the STD-C and CAF-C groups received only the vehicle daily, and the rest of the groups received the corresponding dose of TetraSOD^®^ per bw mixed with the same volume of vehicle. Four days before the beginning of the treatments, the rats were trained to lick diluted low-fat condensed milk (0.3 mL) to ensure voluntary consumption [38]. Considering that the CAF rat’s average weight was 540 g from weeks 9 to 16, the doses of TetraSOD^®^ of 0.17, 1.7 and 17 mg kg^−1^ bw were equivalent to a daily consumption of 2.5, 25 and 250 mg of TetraSOD^®^, respectively, for a 60 kg human [39]. The dose of 2.5 mg/day was chosen as lower than that evaluated in previous clinical studies [40], the dose of 25 mg/day corresponds to a dose that has previously shown positive effects in humans [40] and the dose of 250 mg/day corresponds to the maximum authorised amount in the European Union that can be used as a food supplement (Regulation EU 2017/2470). Body weight was determined once per week. Food intake was determined once per week during a 24 h period, and food was renewed daily. The day of food intake control, each item of the diet was weighed separately and placed in the cage with new sawdust. After 24 h, the final weight of each element of the diet was measured to calculate the amount consumed. At the 15th week, blood samples were also obtained for the evaluation of biochemical parameters by saphenous vein puncture. After immobilizing the rat, a puncture was made in the saphenous vein with a 21G needle, and the emanated blood was collected using tubes containing heparin.

### 2.3. Body Composition Analyses

Lean and fat mass measurements were performed without anaesthesia on the first day of the study, at the end of the 8th week (before the beginning of the TetraSOD^®^ treatment), and during the 16th week (at the end of the treatment) by proton nuclear magnetic resonance (NMR) using an EchoMRI-700™ device (Echo Medical Systems, LLC, Houston, TX, USA). The measurements were performed in triplicate, in *ad libitum* conditions and at 8.00 A.M. The method creates contrasts between soft tissues by exploiting differences in the relaxation times of hydrogen proton spins in different environments. Radio pulses cause the spins of protons to process and emit radio signals. These signals are then received and analysed. The amplitude, duration, and spatial distribution of these signals were determined by the properties of the material scanned. The high contrast between fat and lean is increased by the use of specially composed radio pulses.

### 2.4. Analysis of Biochemical Parameters in Plasma

Blood samples were obtained by saphenous vein puncture and collected in heparinized tubes at the end of the 8th week (before treatment) and 16th week (during treatment). To obtain plasma samples, blood was centrifuged for 10 min at 12,000× *g* at 4 °C. The circulating levels of total cholesterol (TC), triacylglycerols (TG), high-density lipoprotein cholesterol (HDL-C), low-density lipoprotein + very low-density lipoprotein cholesterol (LDL/VLDL-C), oxidized-LDL (oxLDL), glucose, insulin and nonesterified free fatty acids (NEFAs) were analysed. Colorimetric kits were used to quantify the circulating levels of glucose, TC and TG (QCA, Amposta, Spain), HDL-C and LDL/VLDL-C (Bioassay Systems, Hayward, CA, USA), and NEFAs (Wako, Neuss, Germany). A quantitative sandwich enzyme immunoassay technique was used to quantify the insulin (Mercodia, Uppsala, Sweden) and oxLDL levels (Cusabio, Houston, TX, USA). From the fasting glucose and insulin values, the homeostatic model assessment insulin resistance (HOMA-IR) index was calculated, which is considered a robust clinical and epidemiological tool for the evaluation of insulin resistance. The HOMA-IR index was calculated by applying the formula proposed by Matthews et al. [41]: [(Glucose (mM) × Insulin (μU/mL)/22.5]. The Quantitative Insulin Sensitivity Check Index (R-QUICKI) as an indicator of insulin sensitivity was calculated using the following formula: 1/[log insulin (μU/mL) + log glucose (mg/dL) + log NEFAs (mmol/L)] [42].

### 2.5. End Point Blood and Tissue Collection

During the 16th week, the rats were sacrificed under anaesthesia (pentobarbital sodium, 80 mg kg^−1^ bw, Merck, Danvers, MA, USA) after 8 h of diurnal fasting. Blood was collected by cardiac puncture, and plasma was obtained by centrifugation and stored at −80 °C until analysis. Liver, white adipose tissue (WAT) depots (retroperitoneal (RWAT), mesenteric (MWAT), epididymal (EWAT) and inguinal (IWAT)), interscapular brown adipose tissue (BAT), skeletal muscles from the right leg (soleus and gastrocnemius), intestines (small and large), caecum, spleen and thymus were rapidly removed, weighed, frozen in liquid nitrogen and stored at −80 °C until analysis.

### 2.6. Analysis of Oxidative Stress and Inflammatory Parameters in Plasma

Circulating levels of the proinflammatory chemokine monocyte chemoattractant protein-1 (MCP-1) and the anti-inflammatory cytokine interleukin-10 (IL-10) were analysed using ELISA kits (Thermo Fisher Scientific, Waltham, MA, USA, and Merck, Danvers, MA, USA, respectively). Circulating levels of malondialdehyde (MDA) were measured by the TBARS method [6] (Cayman, Hamburg, Germany) as a marker of lipid peroxidation. Circulating levels of nitric oxide (NOx) metabolites (nitrite/nitrate) were evaluated as a marker of oxidative stress by a colorimetric assay (Cayman, Hamburg, Germany) [43].

### 2.7. Analyses in Liver

Levels of SOD (Thermo Fisher Scientific, Waltham, MA, USA), CAT and GPx (Abcam, Cambridge, UK), GSH (BioVision, San Francisco, CA, USA) and MDA (Cayman, Hamburg, Germany) were determined by ELISA kits following the manufacturer’s instructions.

### 2.8. Analyses in WAT

The adiposity index was computed for each animal as the sum of the EWAT, IWAT, MWAT and RWAT depot weights and were expressed as a percentage of the total body weight.

### 2.9. Total RNA Isolation and cDNA Synthesis

Total RNA from the liver, MWAT, thymus, and spleen was isolated using TRIsure™ (Meridian Bioscience, Memphis, TN, USA) following the instructions provided by the manufacturer. Briefly, 1 mL was added to 50–60 mg of each tissue sample in a Lysing Matrix D tube (MP Biomedicals, LLC, Irvine, CA, USA), and thereafter, samples were homogenized using a FastPrep-24™ instrument (MP Biomedicals, LLC, Irvine, CA, USA) set at 6 m s^−1^ for 40 s. To ensure complete disruption of the tissue samples, three lysis cycles using the same settings were applied. Then, RNA was purified using the ISOLATE II RNA Mini Kit (Meridian Bioscience, Memphis, TN, USA) following the manufacturer’s instructions. RNA samples were treated twice with DNase I to avoid further PCR amplification of residual traces of genomic DNA. Finally, all RNA samples were quantified using a NanoDrop 2000 spectrophotometer (Thermo Fisher Scientific, Waltham, MA, USA). The quality of the RNA was checked by agarose gel electrophoresis.

For cDNA construction, 1 µg of RNA was reverse-transcribed using the iScript™ cDNA Synthesis Kit (Bio-Rad, Hercules, CA, USA) in a 20 µL reaction volume according to the manufacturer’s instructions. Thereafter, the reactions were diluted 5-fold by the addition of 80 µL nuclease-free water. In each tissue, two randomly selected RNA samples were used as a control to confirm the absence of genomic DNA contamination by direct amplification of RNA in the absence of cDNA synthesis.

### 2.10. Primer Design and RT-qPCR Synthesis

Specific primer pairs (Table 2) were designed for all target genes using Oligo v7.60 software (Molecular Biology Insights, Cascade, CO, USA) and then ordered from a commercial supplier (Thermo Fisher Scientific, Waltham, MA, USA). The appropriate performance of the primer pairs was checked by PCR amplification using the same conditions described below for RT-qPCR and further melting curve analysis from 65 to 95 °C with a ramp speed of 0.5 °C every 10 s. In all instances, single and sharp peaks were obtained. Moreover, the amplified products were analysed by standard agarose gel electrophoresis (2.5% in TAE 1X) to confirm the presence of single DNA bands of the expected size.

RT-qPCR was conducted using a CFX96™ Real-Time PCR Detection System (Bio-Rad, Hercules, CA, USA). Reactions were performed in a final volume of 10 µL containing 5 µL of 2X SsoAdvanced Universal SYBR^®^ Green Supermix (Bio-Rad, Hercules, CA, USA), 300 nM of both forward and reverse primers (0.3 µL of a 10 µM stock each), 2 µL of cDNA (corresponding to the cDNA retrotranscribed from 20 ng of RNA), and 2.4 µL of nuclease-free water. The thermal cycling profile included an initial incubation at 95 °C for 30 s, followed by 40 cycles of 95 °C for 15 s and 60 °C for 15 s. Reactions were run in duplicate, and the mean threshold cycle (Ct) was further employed to determine relative transcript levels according to the 2^−∆∆Ct^ method [44]. The geometric average of three reference genes (RPLP1, PPIA, and SDHA in liver; RPLP1, PPIA, and B2 M in MWAT; RPLP1, PPIA, and RPL32 in thymus and spleen) was employed for gene expression normalization according to the outputs of both geNorm [45] and NormFinder [46] analyses.

### 2.11. Statistical Analyses

Statistical analyses were performed using the IBM SPSS Statistics 25.0 program. Grubbs’ test was used to detect outliers, which were discarded from subsequent analyses. The assumption of normality was determined using the Kolmogorov–Smirnov test, and the homoscedasticity among groups was assessed using Levene’s test. When one or both of these conditions were not met, data were transformed to a base-10 logarithm to obtain a normal distribution. Differences in the morphometric and biochemical parameters between rats fed the STD diet (*n* = 10) and rats fed the CAF diet prior to treatment (*n* = 40) were detected by the Mann–Whitney U nonparametric test for independent samples. Differences in the STD-C (*n* = 10) vs. CAF-C (*n* = 10) groups during the treatment period and at the end point of the study were assessed using Student’s t parametric test (*t* test) for independent samples. One-way ANCOVA followed by Duncan’s post hoc test was used to determine significant differences among the four groups that received the CAF diet, using the percentage of body fat in the 8th week (before treatment) as a covariable to minimize the variability in the results derived from the diet that could mask possible subtle effects of the treatment. When significant differences were not obtained by comparing the four groups at once, a pairwise analysis was also performed comparing each treatment with respect to the CAF-C group, using ANCOVA and considering the percentage of body fat in the 8th week as covariable.

Data are presented as the mean ± standard error of the mean (SEM). The statistical significance level was set at bilateral 5% (*p* < 0.05), and 0.05 < *p* < 0.1 was considered to indicate a tendency towards significance.

## 3. Results

### 3.1. CAF Intake Induces MetS-Like Alterations

The consumption of the CAF diet for 8 weeks induced most of the alterations that characterize MetS in rats (Table 3), including increased body weight and fat mass, hyperglycaemia, hyperinsulinaemia, insulin resistance, hypertriglyceridemia and chronic low-grade systemic inflammation. Increased caloric intake was observed in CAF-fed animals compared with STD-fed rats due to a higher cumulative carbohydrate and fat intake, which would explain, at least in part, these deleterious effects attributed to the CAF diet. Additionally, a significant decrease in the relative lean mass content as well as in the lean/fat ratio was observed in CAF-fed animals compared with STD-fed rats, an effect that could be partially explained by the decreased cumulative protein intake observed in CAF-fed animals. These deleterious effects remained until the end of the study (Table 4). Increased body weight and fat mass would be explained by a higher visceral mass index in CAF-fed animals because of an increase in the weight of adipose tissues. An increase in the weight of adipose tissue depots and consequently a higher visceral mass index in the liver and thymus, a lower weight of the caecum, a greater length of the small intestine, and a trend towards decreased colon length were also detected in CAF-fed animals when compared to the STD-fed rats. In addition, at the end of the study, CAF-C rats also showed a decrease in IL-10 plasma levels (Table 4).

At the end of the study, no significant changes in body composition, including body weight, fat and lean mass percentages, lean/fat rate, adiposity index and tissue weight, were observed after supplementation with TetraSOD^®^ compared to animals fed the CAF diet (Table 4). In addition, no changes were observed in the dietary parameters associated with the consumption of TetraSOD^®^ (Table 4). Although no differences were observed in the length of the colon, when comparing all of the groups fed the CAF diet, the pairwise comparisons showed that the length of the colon in the CAF + 17 group was significantly shorter (*p* = 0.032), and a downwards trend in the CAF + 1.7 group (*p* = 0.096) was observed compared to CAF-C rats.

Supplementation with TetraSOD^®^ resulted in no significant changes in insulin levels or insulin resistance and sensitivity, as measured by the HOMA-IR and R-QUICKI indices, respectively, in CAF-fed rats compared to those supplemented with the vehicle. However, circulating glucose plasma levels (*p* = 0.095) tended to decrease in the CAF + 17 group compared to the CAF-C group, after pairwise comparisons were performed (Table 4). No significant changes in TC, HDL-C, TG and NEFA levels were observed between CAF-fed rats that received TetraSOD^®^ or vehicle (Table 4). Nevertheless, pairwise comparisons revealed that circulating levels of LDL/VLDL-C tended to be reduced (*p* = 0.092) in the CAF + 0.17 group compared to the group that received only the vehicle (Table 4).

### 3.2. TetraSOD^®^ Supplementation Modulates Oxidative Stress and Inflammatory Markers in the Plasma of CAF-Fed Rats

Although no clear effect of the diet on oxLDL or NOx levels was observed, an antioxidant effect of TetraSOD^®^ was detected in the CAF + 17 obese rats, decreasing oxLDL circulating levels (−11.89%) compared to animals fed a CAF diet and vehicle (*p* = 0.044) after pairwise comparisons were performed (Table 3). Plasma NOx levels were enhanced in CAF + 0.17 rats (+56.67%) in comparison to the CAF-C group (Table 4). Moreover, an anti-inflammatory effect with increasing IL-10 levels in plasma was observed in the CAF + 17 group in contrast to the CAF-C group (+113%) (Table 4).

### 3.3. TetraSOD^®^ Supplementation Promotes Antioxidant Enzymes and GSH in the Livers of CAF-Fed Rats

**Figure 1:**
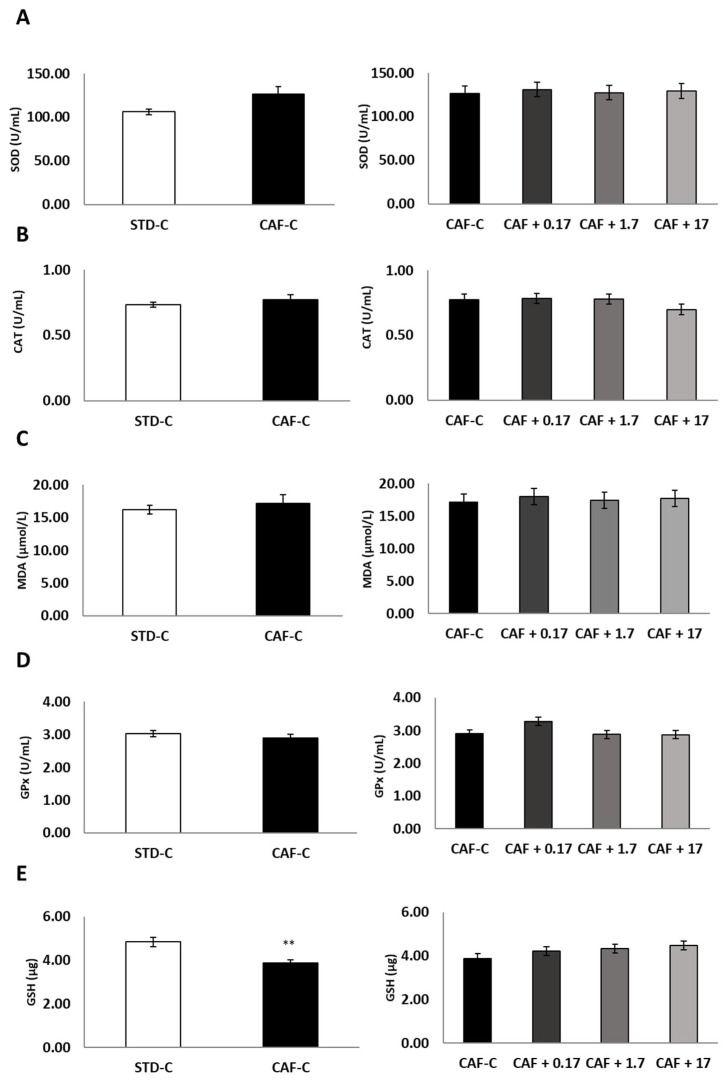
Hepatic SOD (**A**), CAT (**B**), MDA (**C**), GPx (**D**) and GSH (**E**) levels. All values are means ± standard error of the mean (SEM). Differences between rats fed standard (STD) diet and supplemented with vehicle (STD-C, *n* = 10) and rats fed cafeteria (CAF) diet and supplemented with vehicle (CAF-C, *n* = 10) were assessed by using Student’s *t* parametric test (*t* test) for independent samples: **, significantly different compared to the STD-C group (*p* < 0.01). CAT, catalase; GPx, glutathione peroxidase; GSH, glutathione; MDA, malondialdehyde; SOD, superoxide dismutase.

Supplementation of CAF-fed rats with TetraSOD^®^ did not affect either MDA levels or CAT and SOD activity in the liver (Figure 1A–C). Even though the CAF diet did not alter the activity of the GPx enzyme, the comparison of the four cafeteria groups revealed a trend towards significance, showing increased GPx activity in the liver (+12.71%) of the CAF + 0.17 animals (Figure 1D). These differences were statistically significant when pairwise analysis was performed to compare the CAF + 0.17 versus CAF-C groups (*p* = 0.043). The CAF diet displayed a deleterious effect on GSH-related antioxidant defences by decreasing the hepatic levels of GSH (Figure 1E). However, an increase in GSH levels could be measured in the CAF + 17 animals (+15.21%) with respect to the CAF-C group after pairwise analysis (*p* = 0.018).

### 3.4. TetraSOD^®^ Positively Modulates Genes Involved in Antioxidant and Anti-Inflammatory Pathways in the Liver

**Figure 2:**
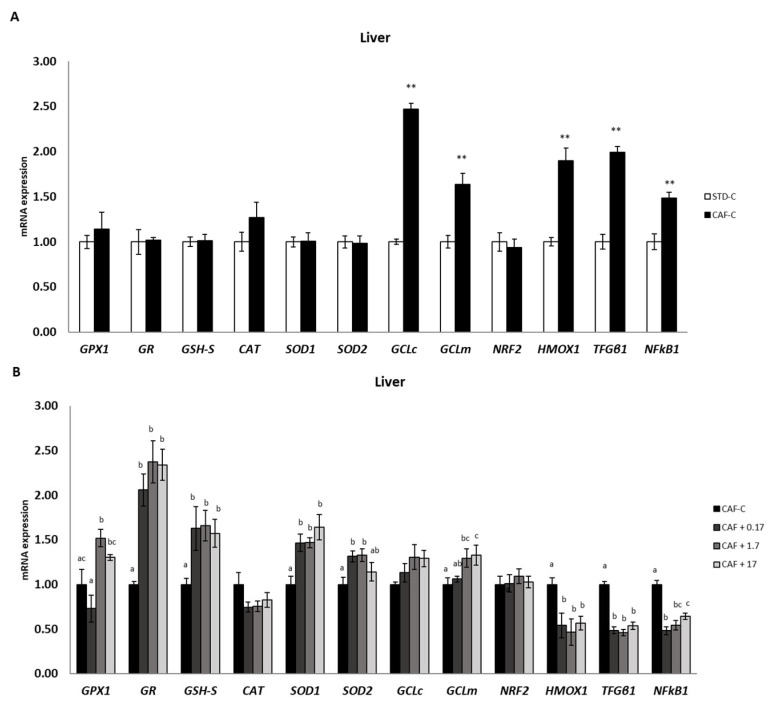
mRNA expression in liver. All values are means ± standard error of the mean (SEM). Differences between rats fed standard (STD) diet and supplemented with vehicle (STD-C, *n* = 8) and rats fed cafeteria (CAF) diet and supplemented with vehicle (CAF-C, *n* = 8) (**A**) were assessed by using Student’s *t* parametric test (*t* test) for independent samples: **, significantly different compared to the STD-C group (*p* < 0.01). Differences among CAF-C rats, rats fed CAF diet and supplemented with 0.17 mg kg^−1^ body weight (bw) per day of TetraSOD^®^ (CAF + 0.17, *n* = 8), rats fed CAF diet and supplemented with 1.7 mg kg^−1^ bw of TetraSOD^®^ (CAF + 1.7, *n* = 8) and rats fed CAF diet and supplemented with 17 mg kg^−1^ bw of TetraSOD^®^ (CAF + 17, *n* = 8) (**B**) were evaluated by the ANCOVA test, using the percentage of body fat in the 8th week (before treatment) as a covariable: ^abc^ letters indicate statistically significant differences among the CAF groups (*p* < 0.05). CAT, catalase; GCLc, glutamate–cysteine ligase, catalytic subunit; GCLm, glutamate–cysteine ligase, modifier subunit; GPx1, glutathione peroxidase 1; GR, glutathione reductase; GSH-S, glutathione synthetase; HMOX1, heme oxygenase 1; NFκB1, nuclear factor kappa B subunit 1; NRF2, nuclear factor, erythroid 2-like 2; SOD1, superoxide dismutase 1; SOD2, superoxide dismutase 2; TGFβ1, transforming growth factor beta 1.

The expression of different GSH-related genes was analysed in the liver (Figure 2). Although no significant differences were found between the STD-C and CAF-C groups, the CAF + 1.7 group displayed a significant upregulation of GPX1 with respect to the CAF-C group. An increase in the mRNA levels of this gene was also observed in the CAF + 17 group in relation to CAF-C, but it was not statistically significant when the analysis was performed including all four groups fed the CAF diet. However, these results were statistically significant when comparing only CAF + 17 with CAF-C animals (*p* = 0.045). Both GR and GSH synthetase (GSH-S) genes exhibited quite similar expression profiles, with significant upregulation in all TetraSOD^®^ supplemented groups in relation to the CAF-C group. SOD1 and SOD2 did not exhibit significant differences in transcripts between the STD and CAF-C groups. However, for both genes, TetraSOD^®^ supplementation induced a significant upregulation in all instances, except for SOD2 in the CAF + 17 group. The expression pattern of the two genes encoding subunits of the glutamate–cysteine ligase, GCLc and GCLm, was affected by CAF and TetraSOD^®^. Although CAF diet induced a significant upregulation in relation to the STD-C group, additional effects derived from TetraSOD^®^ supplementation were only observed in GCLm. In this regard, a significant increase in mRNAs was detected in the CAF + 1.7 and CAF + 17 groups when compared to the CAF-C group.

Among the analysed genes playing a known role in anti-inflammatory pathways, no significant differences were observed for NRF2 among dietary treatments, whereas HMOX1, TGFβ1 and NFκB1 showed a significant upregulation as a consequence of CAF diet intake. In all instances, TetraSOD^®^ induced a further significant downregulation with regard to the CAF-C group.

### 3.5. TetraSOD^®^ Positively Modulates Genes Involved in Anti-Inflammatory Pathways in MWAT

**Figure 3:**
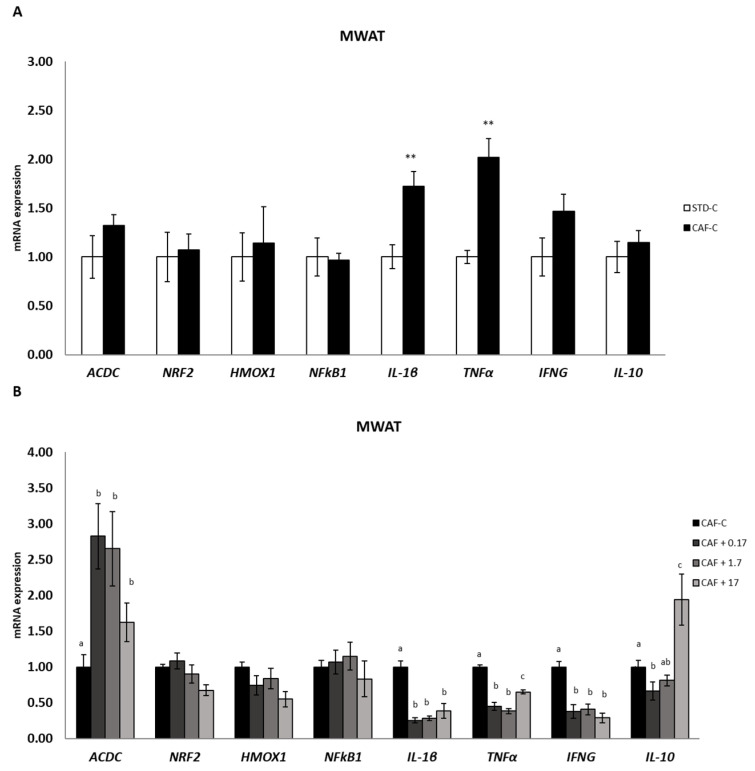
mRNA expression in MWAT. All values are means ± standard error of the mean (SEM). Differences between rats fed standard (STD) diet and supplemented with vehicle (STD-C, *n* = 8) and rats fed cafeteria (CAF) diet and supplemented with vehicle (CAF-C, *n* = 8) (**A**) were assessed by using Student’s *t* parametric test (*t* test) for independent samples: **, significantly different compared to the STD-C group (*p* < 0.01). Differences among CAF-C rats, rats fed CAF diet and supplemented with 0.17 mg kg^−1^ body weight (bw) per day of TetraSOD^®^ (CAF + 0.17, *n* = 8), rats fed CAF diet and supplemented with 1.7 mg kg^−1^ bw of TetraSOD^®^ (CAF + 1.7, *n* = 8) and rats fed CAF diet and supplemented with 17 mg kg^−1^ bw of TetraSOD^®^ (CAF + 17, *n* = 8) (**B**) were evaluated by the ANCOVA test, using the percentage of body fat in the 8th week (before treatment) as a covariable: ^abc^ letters indicate statistically significant differences among the CAF groups (*p* < 0.05). ACDC, adiponectin; HMOX1, heme oxygenase 1; IFNG, interferon gamma; IL-1β; interleukin 1 beta; IL-10; interleukin 10; NFκB1, nuclear factor kappa B subunit 1; NRF2, nuclear factor, erythroid 2-like 2; TNFα, tumour necrosis factor alpha.

The expression profile of the gene encoding adiponectin (ACDC) was first analysed. No significant differences could be detected in the CAF-C group compared to the STD-C group. However, an increase in transcripts was detected in all TetraSOD^®^-supplemented groups, although only in the CAF + 0.17 and CAF + 1.7 rats was such an increase significant (Figure 3). Whereas NRF2, HMOX1 and NFκB1 did not exhibit significant differences among the dietary interventions, genes encoding the proinflammatory cytokines IL-1β and TNFα exhibited a significant increase in transcripts in the CAF-C group when compared to the STD-C group. However, TetraSOD^®^ supplementation induced a significant downregulation in relation to the CAF-C group in both genes. In addition, although no significant differences in transcripts were detected in the pro-inflammatory IFNG between the STD-C and CAF-C groups, TetraSOD^®^ induced a significant downregulation in all supplemented groups.

No significant differences were found in the anti-inflammatory IL-10 gene between the STD-C and CAF-C groups. A significant decrease in the mRNA levels of this gene was observed in CAF + 0.17 rats when compared to the CAF-C group, but in contrast, a significant upregulation was detected in the CAF + 17 group.

### 3.6. TetraSOD^®^ Modulates Genes Involved in Immunity and Anti-Inflammatory Pathways in the Thymus

**Figure 4:**
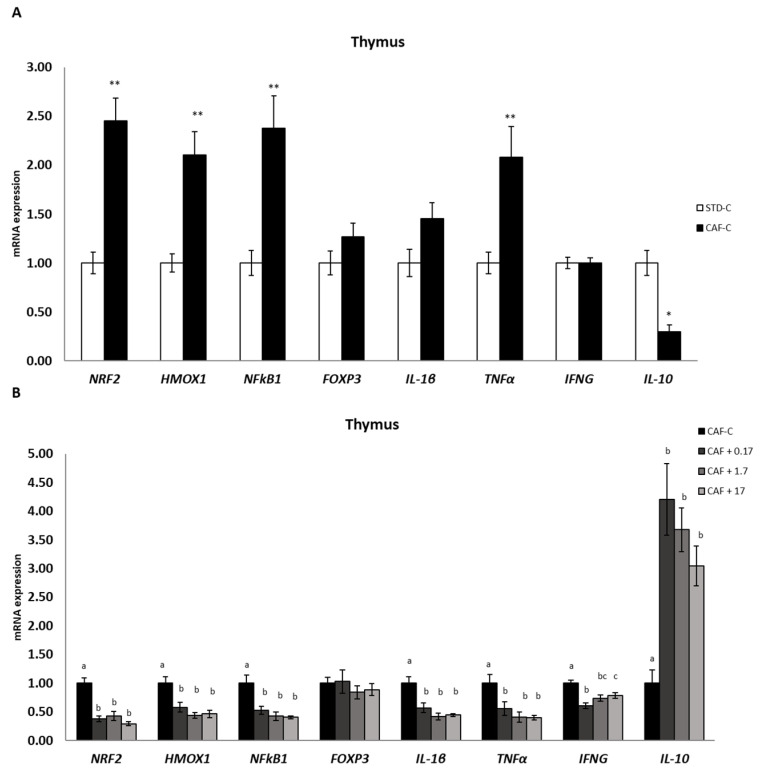
mRNA expression in the thymus. All values are means ± standard error of the mean (SEM). Differences between rats fed standard (STD) diet and supplemented with vehicle (STD-C, *n* = 8) and rats fed cafeteria (CAF) diet and supplemented with vehicle (CAF-C, *n* = 8) (**A**) were assessed by using Student’s *t* parametric test (*t* test) for independent samples: *, significantly different compared to the STD-C group (*p* < 0.05); **, significantly different compared to the STD-C group (*p* < 0.01). Differences among CAF-C rats, rats fed CAF diet and supplemented with 0.17 mg kg^−1^ body weight (bw) per day of TetraSOD^®^ (CAF + 0.17, *n* = 8), rats fed CAF diet and supplemented with 1.7 mg kg^−1^ bw of TetraSOD^®^ (CAF + 1.7, *n* = 8) and rats fed CAF diet and supplemented with 17 mg kg^−1^ bw of TetraSOD^®^ (CAF + 17, *n* = 8) (**B**) were evaluated by the ANCOVA test, using the percentage of body fat in the 8th week (before treatment) as a covariable: ^abc^ letters indicate statistically significant differences among the CAF groups (*p* < 0.05). FOXP3, forkhead box P3; HMOX1, heme oxygenase 1; IFNG, interferon gamma; IL-1β; interleukin 1 beta; IL-10; interleukin 10; NFκB1, nuclear factor kappa B subunit 1; NRF2, nuclear factor, erythroid 2-like 2; TNFα, tumour necrosis factor alpha.

In the thymus (Figure 4), a significant increase in TNFα, NRF2, HMOX1 and NFκB1 transcripts was detected in the CAF-C rats in relation to the STD-C group. An increase in transcripts, but not quite significantly significant (*p* = 0.051), was also detected in IL-1β between the CAF-C and STD-C groups, whereas the same transcript levels were observed in IFNG between those groups. TetraSOD^®^ counteracted the increased expression of these genes induced by consumption of the CAF diet. The three doses of TetraSOD^®^ downregulated all of these genes with respect to the CAF-C group. The expression profile of the anti-inflammatory IL-10 gene was quite different, as a significant downregulation was observed in the CAF-C group in relation to the STD-C group, and all TetraSOD^®^ doses induced a significant upregulation.

### 3.7. TetraSOD^®^ Modulates Genes Involved in Immunity and Anti-Inflammatory Pathways in the Spleen

**Figure 5:**
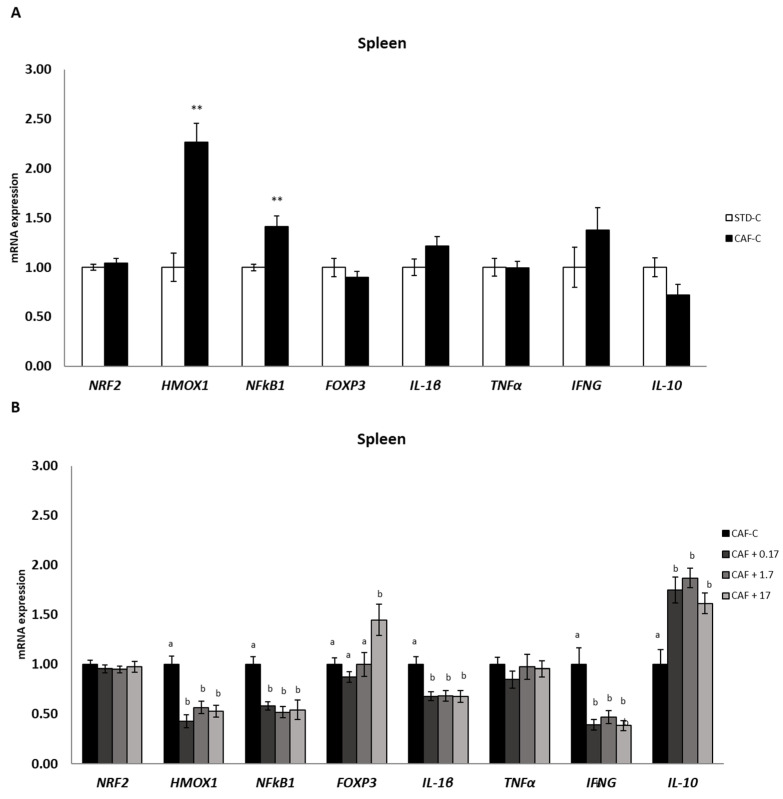
mRNA expression in the spleen. All values are means ± standard error of the mean (SEM). Differences between rats fed standard (STD) diet and supplemented with vehicle (STD-C, *n* = 8) and rats fed cafeteria (CAF) diet and supplemented with vehicle (CAF-C, *n* = 8) (**A**) STD-C were assessed by using Student’s *t* parametric test (*t* test) for independent samples: **, significantly different compared to the STD-C group (*p* < 0.01). Differences among CAF-C rats, rats fed CAF diet and supplemented with 0.17 mg kg^−1^ body weight (bw) per day of TetraSOD^®^ (CAF + 0.17, *n* = 8), rats fed CAF diet and supplemented with 1.7 mg kg^−1^ bw of TetraSOD^®^ (CAF + 1.7, *n* = 8) and rats fed CAF diet and supplemented with 17 mg kg^−1^ bw of TetraSOD^®^ (CAF + 17, *n* = 8) (**B**) were evaluated by the ANCOVA test, using the percentage of body fat in the 8th week (before treatment) as a covariable: ^ab^ letters indicate statistically significant differences among the CAF groups (*p* < 0.05). FOXP3, forkhead box P3; HMOX1, heme oxygenase 1; IFNG, interferon gamma; IL-1β; interleukin 1 beta; IL-10; interleukin 10; NFκB1, nuclear factor kappa B subunit 1; NRF2, nuclear factor, erythroid 2-like 2; TNFα, tumour necrosis factor alpha.

In this secondary lymphoid tissue (Figure 5), a significant upregulation of FOXP3 was observed in CAF + 17 rats relative to the CAF-C group. Moreover, no significant differences were detected between dietary treatments in NRF2 or the proinflammatory gene TNFα. Although no significant differences could be observed between the CAF-C and STD-C groups in other proinflammatory genes such as IL-1β and IFNG, a significant downregulation of these two genes was detected after TetraSOD^®^ supplementation at all doses. In addition, as observed in the thymus, HMOX1 and NFκB1 were significantly upregulated in CAF-C animals in relation to the STD-C group, and all TetraSOD^®^ doses induced a significant downregulation when compared to the CAF-C group. No significant changes in transcripts between the CAF-C and STD-C groups were observed for IL-10, which was upregulated in all TetraSOD^®^ supplemented groups in relation to the CAF-C group.

## 4. Discussion

In the present study, our findings show subtle effects of TetraSOD^®^ in metabolic parameters, tending to decrease plasma glucose after 17 mg kg^−1^ bw of TetraSOD^®^ administration and circulating levels of LDL/VLDL-C in the CAF + 0.17 group, compared to the group that received CAF diet and the vehicle. After 8 weeks of nutritional intervention with the CAF diet, clear metabolic alterations that characterize MetS were observed in our rats, including increased body weight and fat mass, hyperglycaemia, hyperinsulinaemia, insulin resistance, hypertriglyceridemia and chronic low-grade systemic inflammation. Following a corrective therapeutic strategy with these doses of TetraSOD^®^ would not be enough to improve advanced obesity and the metabolic alterations associated with MetS that was achieved after 8 weeks of CAF diet. However, testing these doses in early stages of obesity or following preventive strategies could be considered for future studies.

Although no clear effect of diet on oxLDL and NOx levels was observed in this study, TetraSOD^®^ showed antioxidant properties in obese rats, decreasing oxLDL circulating levels in the CAF + 17 group and enhancing NOx in plasma in the CAF + 0.17 rats compared to control animals, two representative parameters of oxidative stress and endothelial dysfunction. oxLDL are particles of circulating LDL that may contain peroxides or their degradation products [47]. The oxidative conversion of LDL to oxLDL is generally considered to be a key event in the biological process that initiates and accelerates the development of early atherosclerotic lesions [48]. In contrast, NOx is produced in the endothelium, and it plays a fundamental role in the normal maintenance of endothelial and vascular function and participates in the inflammatory response [49]. However, in a state of inflammation, NOx production increases considerably, contributing to oxidative stress [50]. There are few studies in which an attenuating effect on lipid oxidation was demonstrated after supplementation with microalgae extracts and their derivatives [51]. However, in vitro investigations have found that SOD can reduce LDL oxidation [52,53] or inhibit oxLDL-induced apoptosis in agreement with our results [54]. Moreover, an increase in NOx levels found after consuming marine components, including microalgae supplementation, has been previously noted. In vitro findings indicated that treatment with an ethanolic extract of *Spirulina* increased NOx production by the endothelium in aortic rings of obese rats [55]. In a prior study, supplementation with a multicomponent produced from the unicellular green alga *Chlorella* lowered arterial stiffness in middle-aged and older persons by enhancing NOx generation by the vascular endothelium [56]. Considering that several steps in the atherosclerotic disease process are inhibited by NOx [57], enhanced endothelial NOx formation by TetraSOD^®^ could be studied in depth as a possible strategy for the management of this disease.

An antioxidant effect in the liver associated with TetraSOD^®^ supplementation was also demonstrated by the promotion of GPx in animals supplemented with 0.17 mg kg^−1^ bw of TetraSOD^®^ Similar findings have been found in obese Golden Syrian hamsters induced by CAF diet consumption and supplemented with melon SOD [7]. In the obese phenotype, there is an imbalance in the normal redox state, which leads to oxidative stress through the synthesis of free radicals and peroxides that damage proteins, lipids and DNA. As mentioned, the enzyme GPx eliminates the reactive species of hydrogen peroxide through the oxidation of GSH to GSH disulfide (GSSG) [58]. However, for the proper maintenance of the oxidative balance of the cell, high levels of the reduced form of GSH and low levels of the GSSG form must be maintained [58]. This tight balance is maintained by the action of the enzyme GSH reductase (GR), which catalyses the reduction of GSSG to GSH [58].

Abnormal blood glucose levels, as seen in the present study, cause liver damage and oxidation, and GSH plays an important role in preventing liver damage under these pathophysiological settings [59]. Thus, an increase in cellular GSH content leads to a considerable decrease in oxidative stress and toxicity [60]. Our results revealed that the CAF diet displayed a deleterious effect on GSH-related antioxidant defences by decreasing the hepatic levels of GSH. However, the supplementation with 17 mg kg^−1^ bw of TetraSOD^®^ increased GSH in liver. GSH is the most important low molecular weight antioxidant synthesized in cells, and numerous biological functions are attributed to it. GSH plays critical roles in protecting cells from oxidative damage and the toxicity of xenobiotic electrophiles and maintaining redox homeostasis [61]. In addition, GSH participates in nutrient metabolism and the regulation of cellular events, including gene expression, DNA and protein synthesis, cell proliferation and apoptosis, signal transduction, cytokine production and immune response [62]. In contrast, GSH deficiency contributes to oxidative stress, which plays a key role in ageing and the pathogenesis of many metabolic diseases, including liver disease, heart disease, obesity and diabetes [62]. Overall, these findings suggest that TetraSOD^®^ could exert its antioxidant properties by inducing endogenous antioxidant defence mechanisms in the liver.

To gain insights into the mechanisms underlying the increase in GSH content in the liver induced by TetraSOD^®^ supplementation, the expression of some of the most relevant genes involved in GSH metabolism was analysed. Specifically, genes encoding the GCL, GSH-S and GR enzymes were selected. Synthesis of GSH occurs directly in the cytosol and involves two consecutive ATP-requiring steps. The first step is the rate-limiting step, in which gamma-glutamylcysteine is formed by the action of GCL, and then GSH-S adds glycine to form the tripeptide gamma-glutamylcysteinylglycine. Moreover, GR catalyses the regeneration of oxidized to reduced GSH [63]. The GCL consists of two subunits, a heavy catalytic subunit (GCLc) and a light regulatory or modifier subunit (GCLm) [64], and the genes encoding both subunits were significantly upregulated in diet-induced obese CAF-fed rats. With regard to the GCLc gene, similar findings were also observed in the liver of obese Zucker rats, as well as in Wistar rats fed a high-cholesterol diet [65,66]. Supplementation with 1.7 and 17 mg kg^−1^ bw of TetraSOD^®^ was able to upregulate the GCLm gene, and a parallel increase in GSH-S transcripts was noted for all three doses. Furthermore, the GR gene was also upregulated by all three TetraSOD^®^ doses. Altogether, the present data strongly suggest that TetraSOD^®^ activates both the de novo synthesis and recycling of GSH.

GPx1 exhibited positive modulation by the two highest doses of TetraSOD^®^ in the liver of obese animals. In vitro, TetraSOD^®^ has also been shown to upregulate GPx1 in human cells [22]. In addition, dietary supplementation with other antioxidant compounds and natural extracts has been demonstrated to upregulate GPx genes in rat hepatic tissue [66,67,68]. Damage at the cellular level by oxidative stress is attenuated by a range of different enzymes, and GPx is one of the most relevant enzymes, being involved in the reduction of hydrogen peroxide to H_2_O oxidizing GSH at the same time. Consequently, the parallel upregulation of genes involved in GSH metabolism as a direct effect of TetraSOD^®^ supplementation might help maintain adequate GSH levels in the liver, protecting cells from oxidative damage [69]. In addition to GPx, the expression of genes encoding the major antioxidant enzymes SOD and CAT was also evaluated in the liver. Although a single gene encodes CAT, up to three different SOD genes have been characterized in mammals, two of them being investigated in the present study: SOD1, which encodes the cytosolic Cu, Zn-SOD, and SOD2, which encodes the Mn-SOD isoform present in mitochondria [70]. In the present study, all TetraSOD^®^ doses significantly increased the expression of the SOD1 gene, whereas the two lowest doses promoted the expression of the SOD2 gene in the liver of diet-induced obese rats. This type of positive response of antioxidant genes to a dietary supplement has been associated with improved antioxidant status [71,72]. A similar response of these two genes to TetraSOD^®^ was also previously observed in an in vitro experimental approach in human myoblasts [22]. Both SOD and CAT activities were elevated by TetraSOD^®^ in exercised muscle tissue in rats [30], suggesting that enhancement of antioxidant activity is the general mechanism underlying the cytoprotective effects of the ingredient.

Nrf2 is a transcription factor that regulates more than 600 genes, including pivotal components in endogenous antioxidant systems [73]. HMOX-1 is a stress-inducible enzyme that is upregulated under the control of Nrf2 by most cell types in response to a wide range of pro-oxidant stimuli, thus providing protection against oxidative damage [74]. Moreover, the Nrf2/HMOX-1 axis also has potent anti-inflammatory properties and immunomodulatory functions [75,76]. Furthermore, NFκB represents a family of inducible transcription factors, in which NFκB1, also known as p50, is one of the representative members involved in many cellular processes, such as cell proliferation and apoptosis, response to infection, oxidative stress response, and inflammation, through the induction of proinflammatory cytokines [77,78]. There is well-known functional crosstalk between the NFκB and Nrf2/HMOX-1 signalling pathways, which interact to control the transcription and function of many downstream target proteins, including inflammatory mediators. This complex interplay, depending on the context and stimuli, results in an enhancement or impairment of the inflammatory response [76,78]. In the experimental model used in this work, transcriptional upregulation of Nrf2 in the thymus as well as HMOX-1 and NFκB1 in the liver, thymus and spleen suggested the existence of a chronic oxidative and proinflammatory stressful status in diet-induced obese rats. Diet supplementation with the three doses of TetraSOD^®^ in obese rats restored the transcript levels in the liver, thymus and spleen of the three genes to similar levels found in animals under the control diet, revealing a potential antioxidant and anti-inflammatory effect of the ingredient.

A significant downregulation of the TGFβ1 gene was measured in all the obese animals supplemented with TetraSOD^®^ in the liver. TGF-β is a pleiotropic cytokine that plays a central role in cell survival, proliferation, differentiation, angiogenesis, and apoptosis [79]. A link between the gene expression profile of inflammatory cytokines, including TGF-β1 and the development of inflammation, was found in adipose and liver tissues in high-fat-fed mice [80]. Thus, the positive effects of TetraSOD^®^ supplementation in the liver, which could derive from the downregulation of the TGF-β1 gene, might be related to an anti-inflammatory effect.

IL-1β and TNF-α are potent pro-inflammatory cytokines expressed in a wide range of tissues and cell types [81,82,83]. In this study, both IL-1β and TNF-α genes were upregulated in the MWAT of obese rats and in the thymus, at levels close to statistical significance, but not in the spleen when compared to healthy animals. Similar results have been previously found in adipose tissue of DIO rats for the TNF-α gene [84,85] and protein [86] or in plasma levels of this cytokine [87,88,89]. Thus, the transcriptional profiles of the TNF-α and IL-1β genes reported in this study agree with the expected inflammatory state characteristics of obesity induced by diet. In animal models of metabolic disorders, the results from different studies suggest that microalgae supplementation is a novel strategy to prevent inflammation given their content in a range of different active ingredients. In this regard, *Phaeodactylum tricornutum*, *Tisochrysis lutea* and *Spirulina platensis* have been shown to be effective in reducing the plasma concentration of TNF-α [88,89] or both TNF-α and IL-1β [90] in DIO rats, and *Odontella aurita* was able to downregulate IL-1β transcripts in the liver to levels measured in healthy animals [91]. In addition, supplementation with *Nannochloropsis gaditana* significantly reduced serum levels of TNF-α in streptozotocin-induced diabetic rats [92]. In the present study, TetraSOD^®^ prompted transcriptional downregulation of both genes at all doses assessed in the MWAT and thymus and significantly reduced IL-1β mRNA levels in the spleen. This protective role seems to be more general, as TetraSOD^®^ was also able to modify the proinflammatory immunoregulatory cytokine profile in rats in response to intense exercise as a way to induce ROS production and inflammation, significantly reducing the levels of TNF-α and IL-1β in serum and muscle [93].

In addition to the previous findings, the three doses of TetraSOD^®^ also elicited a significant downregulation of the IFNG gene in CAF-fed animals in the MWAT, thymus and spleen. IFNG is one of the most important cytokines mediating systemic inflammation in obesity [94,95,96,97]. The results of the present work demonstrate negative transcriptional regulation of the IFNG gene in adipose and immune tissues in animals supplemented with TetraSOD^®^, together with the parallel expression profiles observed for other genes encoding key cytokines regulating the inflammatory response, such as IL-1β and TNF-α, strengthening the relevance of TetraSOD^®^ as a dietary supplement to control and modulate the inflammatory status associated with obesity and MetS.

TetraSOD^®^ supplementation prompted positive regulation with regard to the pleiotropic anti-inflammatory cytokine IL-10. An increase in plasma levels of this cytokine in animals supplemented with the highest dose of TetraSOD^®^ was observed, reaching similar levels to those observed in healthy animals. At the transcriptional level, upregulation of the IL-10 gene was also detected in MWAT at the highest dose. Nevertheless, a more sensitive transcriptional response could be found in immune organs such as the thymus and spleen, with higher IL-10 mRNA amounts in TetraSOD^®^-supplemented animals at all tested doses. These expression patterns support our findings regarding a parallel downregulation of both IL-1β and TNF-α, as IL-10 is known to inhibit the production of both cytokines at the transcriptional level [98,99] by blocking the nuclear localization of NFκB, which is involved in the expression of inflammatory cytokine genes [100]. In HF-fed obese rats, dietary supplementation with the microalga *Tisochrysis lutea* elicited an increase in IL-10 production in abdominal adipose tissue, improving the inflammatory status [93]. Altogether, the reported data postulate TetraSOD^®^ as a therapeutic natural ingredient to fight against low-grade inflammatory detrimental effects caused by obesity via upregulation of IL-10.

Moreover, as IL-10 is known to be involved in T-cell maturation [101], TetraSOD^®^ could act as modulator of the immune response. In this regard, in the spleen, with the highest TetraSOD^®^ dose, a significant upregulation of the Foxp3 gene, which is a pivotal transcriptional regulator in T_reg_ cells controlling their immunosuppressive activity, was observed [102]. A link between obesity and immunological disturbances exists, exerting a negative effect on the primary and secondary responsiveness of lymphoid organs such as the thymus and spleen [103]. Obesity alters the structural features of both the thymus and spleen in obese animals, thus compromising the generation of naïve T cells [104,105]. Moreover, it was found that DIO rats exhibited a decreased immune response, with a reduction in the size of the T-helper pool and an impairment of splenocyte and monocyte responsiveness [106]. Thus, the present findings suggest that TetraSOD^®^ is able to modulate the immune response of obese animals through transcriptional activation of the IL-10 and Foxp3 genes in immune tissues.

Adipose tissue is considered an endocrine organ that produces a number of different biologically active adipokines, which regulate appetite, insulin sensitivity, and inflammation [107]. Among them, ACDC has been shown to exhibit anti-diabetic, anti-atherogenic, and anti-inflammatory effects, and it is also an insulin sensitizer, thus revealing it as a novel therapeutic factor to be targeted for diabetes and MetS [108]. Significant negative regulation of the ACDC gene expression has been found in the WAT of mice fed a HF diet [109]. Our results in the MWAT suggested that the three doses of TetraSOD^®^ could play a protective role against inflammation upregulating the expression of the ACDC gene.

It has been demonstrated in obese mice that ACDC exerts a negative effect on IFNG production by CD4+ T cells [110], which might explain the link between the ACDC and IFNG gene expression patterns in response to TetraSOD^®^ and, by extension, to the gene expression profiles of the additional cytokines addressed in the present study. In this regard, ACDC is also known to induce the production of the anti-inflammatory IL-10 in macrophages and dendritic cells and to suppress the proinflammatory TNF-α in stimulated macrophages, and at the same time, TNF-α can suppress the transcription of the ACDC gene in an adipocyte cell line [111]. That is, it becomes clear that complex regulatory crosstalk controls anti- and proinflammatory cytokine production in response to varying physiological conditions. The results obtained in the present work have demonstrated that this network can be modulated by TetraSOD^®^ to foster an anti-inflammatory state in different organs and tissues.

## 5. Conclusions

In summary, chronic supplementation with TetraSOD^®^ ameliorates oxidative stress and inflammation disturbances associated with MetS. Its beneficial effects against this metabolic disorder include (i) the promotion of endogenous antioxidant defence mechanisms in the liver, (ii) the modulation of oxidative stress and inflammatory markers in plasma, and (iii) the positive modulation of genes involved in antioxidant, anti-inflammatory and immune pathways in the liver, MWAT, thymus and spleen. Altogether, TetraSOD^®^ is postulated to be a promising therapeutic strategy for MetS management.

## Figures and Tables

**Table 1:** Caloric distribution of the diets.

	STD Diet	CAF Diet
Protein (%)	20	10
Fat (%)	13	31.9
Saturated fat (%)	1.9	14.4
Carbohydrate (%)	67	58.1
Sugar (%)		39.8

**Table 2:** List of primers used for RT-qPCR.

Gene Symbol	Gene Name	Primer Sequence	ENSEMBL Accession Number	Amplicon Length (bp)	Optimal T_a_ (°C)
ACDC	Adiponectin	F: 5′-ATTATGACGGCAGCACTGGCAA-3′	ENSRNOG00000001821.6	149	59.2
		R: 5′-TTCCTGATACTGGTCGTAGGTGA-3′			
FOXP3	Forkhead box P3	F: 5′-CTCTGCACCTTCCCACGCTCA-3′	ENSRNOG00000011702.6	142	62.9
		R: 5′-GAAACTCTCCCGGCTCCTCGAA-3′			
GCLc	Glutamate–cysteine ligase, catalytic subunit	F: 5′-GGAGCGAGATGCCGTCTTACAGG-3′	ENSRNOG00000006302.4	102	60.2
		R: 5′-GAGCTGGTCTGGGCCTTGCTA-3′			
GCLm	Glutamate–cysteine ligase, modifier subunit	F: 5′-CACAATGACCCAAAAGAACTGCTC-3′	ENSRNOG00000013409.4	124	60.3
		R: 5′-TCACGATGACCGAGTACCTCAGC-3′			
GPx1	Glutathione peroxidase 1	F: 5′-CGTCCCTCGGCACCACG-3′	ENSRNOG00000048812.2	68	61.3
		R: 5′-ACGAGGCCCCAGACGCTT-3′			
GR	Glutathione-disulfide reductase	F: 5′-CACTTCTCACCCCAGTTGCGA-3′	ENSRNOG00000014915.6	103	60.2
		R: 5′-ACGGTAGGGATGTTGTCATAGTCCA-3′			
GSH-S	Glutathione synthetase	F: 5′-CGCCTTCCTGGAGCAAACACT-3′	ENSRNOG00000018964.2	125	59.8
		R: 5′-GATTGAGGCCCAGGAACACAGT-3′			
HMOX1	Heme oxygenase 1	F: 5′-GACCGCCTTCCTGCTCAACA-3′	ENSRNOG00000014117.7	103	60.6
		R: 5′-CTGGCGAAGAAACTCTGTCTGTGA-3′			
IFNG	Interferon gamma	F: 5′-GACAACCAGGCCATCAGCAACAAC-3′	ENSRNOG00000007468.2	118	58.7
		R: 5′-TCACCTCGAACTTGGCGATGCTCA-3′			
IL-1β	Interleukin 1 beta	F: 5′-CTACCTATGTCTTGCCCGTGGA-3′	ENSRNOG00000004649.4	124	59.9
		R: 5′-CATCACACACTAGCAGGTCGTC-3′			
IL-10	Interleukin 10	F: 5′-TGGCCCAGAAATCAAGGAGCATT-3′	ENSRNOG00000004647.5	116	59.7
		R: 5′-CCACTGCCTTGCTTTTATTCTCACA-3′			
NRF2	Nuclear factor, erythroid 2-like 2	F: 5′-TGCTGCCATTAGTCAGTCGCTCT-3′	ENSRNOG00000001548.6	104	60.4
		R: 5′-CCGTGCCTTCAGTGTGCTTC-3′			
NFkB1	Nuclear factor kappa B subunit 1	F: 5′-GAAGATGTGGTGGAGGACTTGCTGA-3′	ENSRNOG00000023258.6	140	61.5
		R: 5′-GCTGCCTTGCTGTTCTTGAGT-3′			
SOD1	Superoxide dismutase 1	F: 5′-ATTAACTGAAGGCGAGCATGGGT-3′	ENSRNOG00000002115.6	137	60.2
		R: 5′-CTCCAACATGCCTCTCTTCATCCG-3′			
SOD2	Superoxide dismutase 2	F: 5′-AGAACCCAAAGGAGAGTTGCTGGA-3′	ENSRNOG00000019048.4	111	60.1
		R: 5′-CCCCAGCCTGAACCTTGGAC-3′			
TGFβ1	Transforming growth factor, beta 1	F: 5′-CTACCAGAAATATAGCAACAATTCCT-3′	ENSRNOG00000020652.4	141	60.0
		R: 5′-AAGCCCTGTATTCCGTCTCCT-3′			
TNFα	Tumour necrosis factor	F: 5′-ATGGGCTGTACCTTATCTACTCC-3′	ENSRNOG00000055156.1	100	59.4
		R: 5′-TATGAAATGGCAAATCGGCTGAC-3′			
B2M	Beta-2 microglobulin	F: 5′-GCCCAACTTCCTCAACTGCTACGTG-3′	ENSRNOG00000017123.5	148	59.5
		R: 5′-AGTGTGAGCCAGGATGTAGAAAGACC-3′			
PPIA	Peptidylprolyl isomerase A	F: 5′-TATCTGCACTGCCAAGACTGA-3′	ENSRNOG00000027864.5	122	59.3
		R: 5′-TGCTGGTCTTGCCATTCCTG-3′			
RPLP1	Ribosomal protein lateral stalk subunit P1	F: 5′-CGGCAGTCCACAACATGGCT-3′	ENSRNOG00000013874.5	100	60.6
		R: 5′-TTGATCTTATCCTCCGTGACCGT-3′			
RPL32	Ribosomal protein L32	F: 5′-CCAAGAAGTTCATCAGGCACCAGT-3′	ENSRNOG00000010746.5	101	59.6
		R: 5′-CTTGAATCTTCTCCGCACCCTGT-3′			
SDHA	Succinate dehydrogenase complex flavoprotein subunit A	F: 5′-TGGACAGAGCCTCAAGTTCG-3′	ENSRNOG00000013331.5	116	60.5
		R: 5′-TGTCATACCGCAGAGATCGTC-3′			

T_a_: Annealing temperature.

**Table 3:** Body composition and dietary and plasma parameters of rats fed a standard (STD) or cafeteria (CAF) diet at the 8th week of the study.

	STD	CAF	*p*
Body composition			
Weight (g)	379.5 ± 6.2	441.1 ± 6.8 *	<0.001
Weight change (g)	124.1 ± 5.4	189.1 ± 6.4 *	<0.001
Fat (%)	3.3 ± 0.5	12.2 ± 0.6 *	<0.001
Lean (%)	92.2 ± 0.5	83.6 ± 0.7 *	<0.001
Dietary parameters			
Cumulative Energy intake (kcal)	536.2 ± 9.1	1130.5 ± 20.4 *	<0.001
Cumulative Protein (g)	32.2 ± 0.5	28.4 ± 0.5 *	0.001
Cumulative Carbohydrates (g)	77.8 ± 1.3	177.9 ± 3.5 *	<0.001
Cumulative Fat (g)	10.7 ± 0.2	34.2 ± 0.6 *	<0.001
Cumulative Fibre (g)	25.4 ± 0.4	8.2 ± 0.4 *	<0.001
Plasma parameters			
TC (mmol/L)	3.1 ± 0.1	2.9 ± 0.1	0.200
HDL-C (mmol/L)	1.8 ± 0.1	1.8 ± 0.0	0.655
LDL/VLDL-C (mmol/L)	1.2 ± 0.1	1.1 ± 0.0	0.409
oxLDL (ngl/mL)	31.9 ± 0.7	30.5 ± 0.5	0.224
TG (mmol/L)	0.7 ± 0.1	2.3 ± 0.1 *	<0.001
NEFA (mmol/L)	0.5 ± 0.0	0.6 ± 0.0	0.275
Glucose (mmol/L)	6.3 ± 0.1	7.3 ± 0.1 *	<0.001
Insulin (pmol/L)	130.8 ± 12.7	366.7± 29.2 *	<0.001
HOMA-IR	5.1 ± 0.7	16.1 ± 1.5 *	<0.001
R-QUICKI	0.3 ± 0.0	0.2 ± 0.0 *	<0.001

All values are means ± standard error of the mean (SEM). Differences between standard (STD, *n* = 10) and cafeteria (CAF, *n* = 40) rats were assessed by using a Mann–Whitney U nonparametric test for independent samples: *, significantly different compared to the STD group (*p* < 0.05). HDL-C, high-density lipoprotein cholesterol; HOMA-IR, homeostatic model assessment of insulin resistance; LDL/VLDL-C, low-density lipoprotein + very low-density lipoprotein cholesterol; NEFA, nonesterified fatty acid; oxLDL, oxidized low-density lipoprotein; R-QUICKI, revised quantitative insulin sensitivity check index; TC, total cholesterol; TG, triacylglycerols.

**Table 4:** Body composition and dietary and biochemical parameters of rats supplemented with vehicle (C) or TetraSOD^®^ at the 16th week of the study in the two different dietary models (STD or CAF).

	STD-C	CAF-C	*p*	CAF + 0.17	CAF + 1.7	CAF + 17	*p*
Body composition							
Weight (g)	420.7 ± 7.6	529.5 ± 18.1 *	<0.001	538.5 ± 20.9	535.8 ± 14.9	520.5 ± 14.5	0.826
Weight change (g)	165.3 ± 6.9	275.9 ± 17.4 *	<0.001	286.2 ± 20.1	283.9 ± 14.5	270.3 ± 13.6	0.782
Fat (%)	4.8 ± 0.7	17.9 ± 1.7 *	<0.001	16.9 ± 2.4	17.5 ± 1.4	16.8 ± 1.7	0.875
Lean (%)	90.9 ± 0.7	78.5 ± 1.7 *	<0.001	79.6 ± 2.3	78.9 ± 1.4	79.7 ± 1.7	0.817
Lean/fat	24.1 ± 4.3	5.1 ± 0.8 *	<0.001	5.9 ± 1.0	4.9 ± 0.5	4.6 ± 0.4	0.249
Adiposity index (%)	3.5 ± 0.3	8.1 ± 0.7 *	<0.001	8.1 ± 0.9	7.9 ± 0.5	7.8 ± 0.6	0.871
RWAT (g)	4.3 ± 0.5	13.8 ± 1.6 *	<0.001	14.9 ± 2.1	13.6 ± 1.1	12.8 ± 1.2	0.312
MWAT (g)	2.8 ± 0.2	8.2 ± 0.8 *	<0.001	8.5 ± 1.3	8.4 ± 0.7	7.7 ± 0.7	0.747
EWAT (g)	4.2 ± 0.4	12.1 ± 1.5 *	<0.001	12.7 ± 1.9	11.4 ± 1.1	11.1 ± 0.7	0.956
IWAT (g)	2.9 ± 0.3	8.4 ± 0.8 *	<0.001	8.4 ± 1.2	8.5 ± 0.7	7.9 ± 0.8	0.881
BAT (g)	0.4 ± 0.0	0.8 ± 0.1 *	<0.001	0.7 ± 0.1	0.8 ± 0.0	0.8 ± 0.1	0.400
Muscle (g)	2.9 ± 0.1	3.1 ± 0.1	0.420	3.1 ± 0.1	3.0 ± 0.1	3.0 ± 0.1	0.937
Liver (g)	11.1 ± 0.5	15.9 ± 0.6 *	<0.001	16.3 ± 0.7	16.8 ± 0.6	14.8 ± 0.5	0.194
Thymus (g)	0.3 ± 0.0	0.6 ± 0.1 *	0.001	0.5 ± 0.0	0.5 ± 0.0	0.5 ± 0.0	0.904
Spleen (g)	0.8 ± 0.0	0.9 ± 0.1	0.170	0.9 ± 0.1	0.9 ± 0.0	0.9 ± 0.0	0.170
Cecum (g)	4.5 ± 0.3	3.7 ± 0.1 *	0.042	4.5 ± 0.3	4.5 ± 0.3	3.7 ± 0.1	0.526
Small intestine (cm)	105.5 ± 2.2	115.2 ± 2.8 *	0.015	112.8 ± 2.6	116.4 ± 2.4	111.6 ± 2.5	0.565
Colon (cm)	15.6 ± 0.5	17.3 ± 0.8	0.085	15.6 ± 0.8	15.6 ± 0.6	14.9 ± 0.6	0.126
Dietary parameters							
Cumulative Energy intake (kcal)	1005.3 ± 16.5	2208.8 ± 88.8 *	<0.001	2236.5 ± 78.5	2280.6 ± 97.3	2158.5 ± 48.6	0.727
Cumulative Protein (g)	60.3 ± 1.0	53.7 ± 2.3 *	0.022	54.2 ± 2.0	54.2 ± 2.1	51.8 ± 1.4	0.813
Cumulative Carbohydrates (g)	145.9 ± 2.4	346.4 ± 9.2 *	<0.001	358.7 ± 13.2	364.8 ± 15.4	342.7 ± 9.0	0.500
Cumulative Fat (g)	20.2 ± 0.3	64.1 ± 2.7 *	<0.001	66.3 ± 2.6	68.5 ± 3.9	65.5 ± 1.5	0.759
Cumulative Fibre (g)	47.7 ± 0.8	14.6 ± 1.5 *	<0.001	14.1 ± 1.2	13.1 ± 1.2	12.9 ± 1.3	0.682
Plasma parameters							
TC (mmol/L)	3.4 ± 0.1	3.0 ± 0.2	0.062	3.1 ± 0.2	3.2 ± 0.1	3.2 ± 0.1	0.611
HDL-C (mmol/L)	1.9 ± 0.1	1.8 ± 0.1	0.363	1.7 ± 0.1	1.7 ± 0.1	1.9 ± 0.1	0.781
LDL/VLDL-C (mmol/L)	1.2 ± 0.1	1.2 ± 0.1	0.724	1.0 ± 0.1	1.2 ± 0.1	1.1 ± 0.0	0.171
oxLDL (ngl/mL)	26.1 ± 1.3	27.0 ± 1.0	0.577	26.5 ± 1.2	25.9 ± 1.0	23.8 ± 1.0	0.166
TG (mmol/L)	1.0 ± 0.1	1.8 ± 0.1 *	<0.001	2.2 ± 0.3	2.0 ± 0.1	2.3 ± 0.2	0.218
NEFA (mmol/L)	0.6 ± 0.0	0.5 ± 0.0	0.813	0.6 ± 0.1	0.5 ± 0.0	0.5 ± 0.0	0.440
Glucose (mmol/L)	5.6 ± 0.2	7.1 ± 0.4 *	0.003	6.4 ± 0.2	6.6 ± 0.3	6.4 ± 0.2	0.292
Insulin (pmol/L)	216.4 ± 17.4	414.3± 55.6 *	0.001	429.8 ± 44.6	368.6 ± 36.7	420.9 ± 51.2	0.755
HOMA-IR	7.5 ± 0.8	18.0 ± 3.0 *	0.008	17.2 ± 2.3	15.3 ± 1.9	16.4 ± 2.3	0.806
R-QUICKI	0.3 ± 0.0	0.2 ± 0.0 *	0.001	0.2 ± 0.0	0.2 ± 0.0	0.2 ± 0.0	0.642
NOx (µmol/L)	8.9 ± 0.4	8.5 ± 0.6 ^a^	0.551	13.3 ± 0.9 ^b^	9.6 ± 0.9 ^ab^	10.1 ± 0.9 ^ab^	0.048
IL-10 (pg/mL)	89.4 ± 18.3	39.4 ± 4.6 * ^a^	0.013	41.8 ± 11.1 ^a^	45.4 ± 10.5 ^a^	84.4 ± 10.5 ^b^	0.018
MCP-1 (ng/mL)	0.1 ± 0.0	0.2 ± 0.0	0.432	0.2 ± 0.0	0.2 ± 0.0	0.2 ± 0.0	0.434

All values are means ± standard error of the mean (SEM). Differences between rats fed standard (STD) diet and supplemented with vehicle (STD-C, *n* = 10) and rats fed cafeteria (CAF) diet and supplemented with vehicle (CAF-C, *n* = 10) were assessed by using Student’s *t* parametric test (*t* test) for independent samples: *, significantly different compared to the STD-C group (*p* < 0.05). Differences among the CAF-C rats, rats fed CAF diet and supplemented with 0.17 mg kg^−1^ body weight (bw) per day of TetraSOD^®^ (CAF + 0.17, *n* = 10), rats fed CAF diet and supplemented with 1.7 mg kg^−1^ bw per day of TetraSOD^®^ (CAF + 1.7, *n* = 10) and rats fed CAF diet and supplemented with 17 mg kg^−1^ bw per day of TetraSOD^®^ (CAF + 17, *n* = 10) were evaluated by the ANCOVA test, using the percentage of body fat in the 8th week (before treatment) as a covariable: ^ab^ letters indicate statistically significant differences among CAF groups (*p* < 0.05). BAT, interscapular brown adipose tissue; EWAT, epididymal white adipose tissue; HDL-C, high-density lipoprotein cholesterol; HOMA-IR, homeostatic model assessment of insulin resistance; IL-10, interleukin-10; IWAT, inguinal white adipose tissue; LDL/VLDL-C, low-density lipoprotein + very low-density lipoprotein cholesterol; MCP-1, monocyte chemoattractant protein-1; MWAT, mesenteric white adipose tissue; NEFA, nonesterified fatty acids; NOx, nitric oxide; oxLDL, oxidized low-density lipoprotein; R-QUICKI, revised quantitative insulin sensitivity check index; RWAT, retroperitoneal white adipose tissue; TC, total cholesterol; TG, triacylglycerol.

## Data Availability

All data supporting the results generated in this study are available upon reasonable request to the corresponding author.
